# p53 limits B cell receptor (BCR) signalling: a new role for *miR-34a* and FOXP1

**DOI:** 10.18632/oncotarget.26376

**Published:** 2018-11-23

**Authors:** Katerina Cerna, Marek Mraz

**Affiliations:** Molecular Medicine, CEITEC Masaryk University, Brno, Czech Republic; Department of Internal Medicine, Hematology and Oncology, University Hospital Brno and Faculty of Medicine MU, Brno, Czech Republic

**Keywords:** miR-34a, DNA damage, BCR signalling, phosphatase, CD22

B Cell Receptor (BCR) signalling is fundamental for the maturation, survival, and proliferation of B cells, and B cell malignancies frequently harbor mutations in this pathway and/or complex deregulation of interconnected signalling [1]. This is underscored by the remarkable clinical effect of inhibitors targeting BCR-associated kinases, especially in chronic lymphocytic leukemia (CLL); however, in CLL the BCR pathway deregulation is not driven by a mutational mechanism. The differences in BCR signalling propensity contribute to variable prognosis in CLL and other "mature" B cell malignancies [2-4]. Interestingly, it is plausible that a normal (or malignant) B cell has to concurrently resolve a situation where its DNA is damaged, leading to p53 stabilization, while a strong pro-proliferative/pro-survival signal is "coming" from its BCR at the same time, due to antigen binding. This should be precisely regulated since a B cell possesses the physiological potential for clonal proliferation, and any unrepaired genetic aberration would greatly increase the risk of a malignancy.

**Figure 1:**
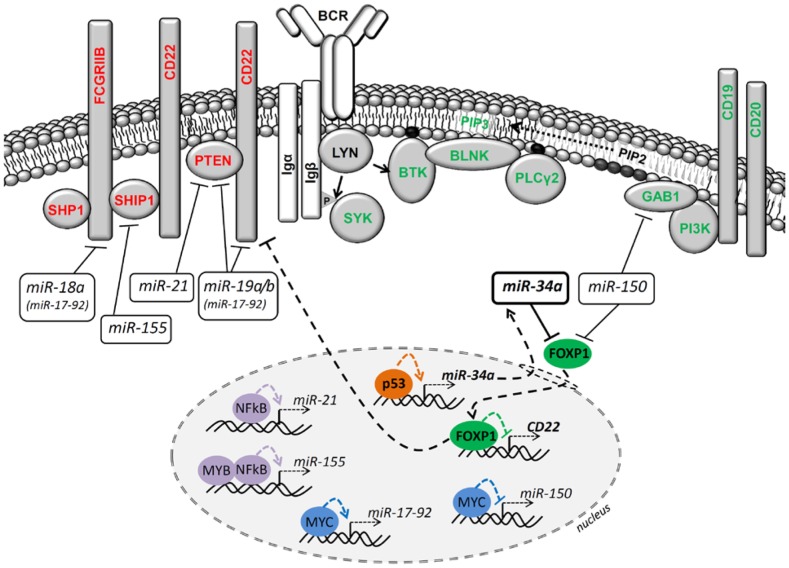
MicroRNAs' contribution to BCR signalling regulation in B cells In B cells, the balance of BCR activation initiation, amplitude and duration can be influenced by a specific immunoglobulin structure, adaptor molecules (e.g. GAB1, BLNK), kinases (e.g. LYN, SYK, BTK, PI3K) or phosphatases (e.g. SHIP1, SHP1 and PTEN) activity and miRNAs levels. The cell-membrane proteins depicted in red represent the "negative" BCR pathway regulators (left part of the figure), and the cell-membrane proteins depicted in green represent the "positive" regulators (right part of the figure). The role of *p53→miR-34a*┤FOXP1 axis during DNA damage response and BCR signalling is highlighted in bold. The regulation of miRNA targets happens at the mRNA stability and/or translation level, which is not visualized here. Direct miRNA transcription regulation by transcription factors in the nucleus is depicted. The sharp point arrow indicates activation; the blunt-end arrow indicates repression.

Indeed, we have observed that when CLL B cells experience DNA damage, their responsiveness to BCR signalling becomes limited, and a similar phenotype can be observed upon forced p53 stabilization by a small molecule Nutlin-3a [5]. We have further described that p53 accumulation induces the microRNA *miR-34a,* which acts as a very potent repressor of a transcription factor FOXP1. FOXP1 is a known positive BCR signalling regulator [2, 5]. Dissecting this observation's consequences requires an understanding of the FOXP1 targets, and multiple studies have identified hundreds of genes that are potentially transcriptionally regulated by FOXP1 [6]. We have shown that in mature B cells, FOXP1 acts as a transcriptional repressor of a cell-membrane molecule CD22, which contains immunoreceptor tyrosine inhibitory motifs and serves as a docking site for phosphatases (Figure 1). The down-modulation of FOXP1 during DNA damage response (DDR) leads to CD22 upregulation, and we suggest that this allows phosphatases such as SHIP1/SHP1 to dock more efficiently to the cell membrane and BCR­ signalosome, and thus limit BCR signalling relatively "upstream" (Figure 1). Altogether, this shows that DDR that does not induce outright cell death leads with some time-delay to signalling repression from the surface BCR. This resembles the situation with p53-mediated signalling inhibition from surface receptors and/or associated kinases observed in unrelated cell types.

We have also shown that clinically used DNA damaging drugs such as fludarabine (or doxorubicin) partially function by inhibiting BCR signalling. The p53 aberrations affect this regulatory mechanism, and the low *miR-34a* levels can serve as an independent predictor of the patient response to chemo-immunotherapy in CLL. We have further developed an assay and determined cut-offs for absolute *miR-34a* quantification using Real-Time PCR to overcome the general limitations of biomarkers based on gene expression levels [5].

The *miR-34a* levels are low in B cells without DDR induction, and thus this miRNA probably only contributes to FOXP1/CD22 regulation when wild-type p53 is accumulated in the cell. However, the p53 defective B cells might also theoretically gain a fitness advantage from higher BCR signalling not only during "drug-induced"

DDR, but also under conditions that involve some level of p53 activity, such as metabolic- or proliferation- induced stress. Nevertheless, we have shown that another miRNA, namely *miR-150,* is mainly responsible for regulating the FOXP1 levels in mature B cells during "basal" conditions not involving DDR [2]. The *miR-150* levels are high in non-diving malignant B cells (one of the most abundant miRNAs), but MYC proto-oncogene leads to its direct repression, subsequent FOXP1 up-regulation and higher BCR signalling propensity [2, 3] (Figure 1). Thus these two miRNAs regulate the same target, but in a different context (basal vs. DDR conditions).

It is unclear if FOXP1 levels affect only the classical "antigen-dependent" signalling or can also change the "tonic" BCR signalling that does not require antigen­ binding and likely depends on the balance of kinases and phosphatases activity on the cell membrane. It is possible that low *miR-150* levels could work as a "tonic" BCR signalling driver via FOXP1 upregulation, and also its other direct target, *GAB1* [2]. GAB1 serves as a docking site for PI3K on the cell membrane, and thus connects the BCR with the amplification of the signal via the PI3K-Akt pathway [1, 2] (Figure 1). It is plausible that a combination of a MYC activating genetic aberration (represses *miR-150)* and p53 deletion/mutation (represses *miR-34a)* could lead to very prominent FOXP1 and BCR signalling upregulation. This might be the case in Richter's transformation of CLL, transformed follicular lymphoma, or *de novo* DLBCL, since these clinically aggressive entities relatively frequently harbor both MYC and p53 aberrations concomitantly.

It has also been shown that other miRNAs, namely *miR-18a* and *miR-19a/b* from the *miR-17-92* cluster, act as repressors of phosphatase-docking molecules *CD22 and FCGR2B* [7], and *miR-19* and *miR-21* (unrelated miRNA) also repress the phosphatase *PTEN* [8, 9] (Figure 1). It has been also shown that *miR-155* (induced by MYB and NFκB signalling) directly represses phosphatase SHIP1 [4, 9] (Figure 1). Notably, the MYC protein induces *miR-17-92* cluster expression in B cells, and the *miR-17-92* is frequently upregulated/amplified in B-NHLs [1]. It has also been demonstrated that forced upregulation of either *miR-155,* the *miR-17-92* cluster, or *miR-19* only leads to a frank B cell lymphoma in mice [8, 9]. Similarly, concurrent *PTEN* and *SHIP1* phosphatases deletion leads to a B cell malignancy in a mouse model [10]. This would indicate that miRNA-mediated changes in the level of "tonic" and/or antigen-induced BCR signalling might be of key importance for the development of a B cell neoplasm; however, a more detailed understanding of miRNA target complexity is needed.

Overall, it is surprising that the connection between p53 and BCR signalling in "mature" malignant B cells has not been investigated in detail previously. It is unlikely that the miR-34a-mediated FOXP1 repression is the only mechanism that p53 utilizes to limit BCR signalling during DDR. Additionally, this process' kinetics during DDR is of interest as well as the potential implications for *miR-34a­* based therapy, which is being considered for clinical trials.
